# A_2_ Adenosine Receptor Subtypes Overproduction in Atria of Perioperative Atrial Fibrillation Patients Undergoing Cardiac Surgery: A Pilot Study

**DOI:** 10.3389/fcvm.2021.761164

**Published:** 2021-11-03

**Authors:** Baptiste Maille, Julien Fromonot, Claire Guiol, Marion Marlinge, Florian Baptiste, Suzy Lim, Charlotte Colombani, Marie Charlotte Chaptal, Mohamed Chefrour, Marguerite Gastaldi, Frederic Franceschi, Jean-Claude Deharo, Vlad Gariboldi, Jean Ruf, Giovanna Mottola, Régis Guieu

**Affiliations:** ^1^Aix Marseille Univ, INSERM, INRAE, C2VN, Marseille, France; ^2^Department of Cardiology, Timone University Hospital, Marseille, France; ^3^Laboratory of Biochemistry, Timone University Hospital, AP-HM, Marseille, France; ^4^Department of Cardiac Surgery, Timone University Hospital, Marseille, France

**Keywords:** atrial fibrillation, adenosine receptor, cardiac surgery, perioperative atrial fibrillation, adenosine receptor 2A, adenosine receptor 2B

## Abstract

**Objective:** Although atrial fibrillation is a common cardiac arrhythmia in humans, the mechanism that leads to the onset of this condition is poorly elucidated. Adenosine is suspected to be implicated in the trigger of atrial fibrillation (AF) through the activation of its membrane receptors, mainly adenosine receptor (AR) subtypes A_1_R and A_2_R. In this study, we compared blood adenosine concentration (BAC), and A_1_R, A_2A_R, and A_2B_R production in right (RA) and left atrium (LA), and on peripheral blood mononuclear cells (PBMCs) in patients with underlying structural heart disease undergoing cardiac surgery with or without peri-operative AF (PeOpAF).

**Methods:** The study group consisted of 39 patients (30 men and 9 women, mean age, range 65 [40–82] years) undergoing cardiac surgery and 20 healthy patients (8 women and 12 men; mean age, range 60 [39–72] years) as controls were included. Among patients, 15 exhibited PeOpAF.

**Results:** Blood adenosine concentration was higher in patients with PeOpAF than others. A_2A_R and A_2B_R production was higher in PBMCs of patients compared with controls and was higher in PeOpAF patients than other patients. In LA and RA, the production of A_2A_R and A_2B_R was higher in patients with PeOpAF than in other patients. Both A_2A_R and A_2B_R production were higher in LA vs. RA. A_1_R production was unchanged in all situations. Finally, we observed a correlation between A_1_R, A_2A_R, and A_2B_R production evaluated on PBMCs and those evaluated in LA and RA.

**Conclusions:** Perioperative AF was associated with high BAC and high A_2A_R and A_2B_R expression, especially in the LA, after cardiac surgery in patients with underlying structural heart disease. Whether these increases the favor in triggering the AF in this patient population needs further investigation.

## Introduction

Although atrial fibrillation is the most common cardiac arrhythmia in clinical practice, the precise mechanism that leads to the onset and persistence of this condition in patients with or without associated structural heart disease has not been fully elucidated.

Adenosine is an ATP derivative released into the extracellular space by endothelial and muscle cells during inflammation (1) or hypoxia (2). Adenosine strongly affects the cardiovascular system *via* four G-protein–coupled adenosine receptors (AR), namely. A_1_R, A_2A_R, A_2B_R, and A_3_R (3, 4). At the cellular level, A_1_R and A_3_R are coupled with Gi-proteins that inhibit cyclic adenosine monophosphate (cAMP) production, whereas A_2A_R and A_2B_R are coupled with Gs-proteins that activate cAMP production in target cells (3, 4).

Adenosine is suspected to be involved in triggering atrial fibrillation (AF) episodes in patients with and without underlying structural heart disease. Indeed, exogenous adenosine administration can provoke spontaneous AF in up to 10% of susceptible patients (5), and there is evidence of a dramatic increase in blood adenosine concentration (BAC) in the left atrium (LA) during episodes of paroxysmal AF in patients without heart structural abnormalities (6). Furthermore, high levels of adenosine plasma were reported in patients with perioperative AF (PeOpAF) during cardiac surgery (7). PeOpAF occurs in 20–50% of patients during cardiac surgery (8, 9) and is often considered a self-limited entity (10, 11). However, the increased risk of long-term stroke and the four to five-fold increased risk of recurrent AF in the next 5 years (12) suggest underlying atrial cardiomyopathy (13), a close mechanism with non-PeOpAF and is a clinically relevant issue (14).

Peripheral blood mononuclear cells (PBMCs) are a good model for the evaluation of adenosine receptor production because the production level of PBMC receptors mirrors their production in cardiovascular tissues (15–17). The aim of this study was to compare adenosine receptor production, i.e., A_1_ R, A_2_A R, and A_2_B R, in atria of patients with structural heart disease undergoing cardiac surgery with or without PeOpAF. We also compared adenosine receptor production by PBMCs to evaluate a possible systemic regulation of the adenosinergic response.

## Methods

### Study Population

We prospectively included patients referred to our center for cardiac surgery from September 2015 to March 2016, and from September 2019 to April 2020. Patients underwent coronary bypass or other cardiac surgery justifying LA atriotomy, under extracorporeal circulation. Transthoracic echocardiography was systematically performed before surgery to assess left ventricular ejection fraction (LVEF), LA enlargement, and underlying valvulopathy. Significant LVEF impairment was considered when LVEF was ≤ 49% (18). Patients < 18 years of age and women who were pregnant or breastfeeding were excluded. The control group comprised healthy subjects who were matched for age and sex with the patient group and were recruited from among the medical staff. Controls were without compromised hemodynamics or underlying suspicion of cardiac abnormality, which were confirmed by normal findings on electrocardiograms and transthoracic echocardiograms, and had no relevant medical history, cardiovascular risk factors, or treatments.

The protocol was approved by the ethics committee of our institution (CPP Sud Méditerranée, Marseille, France, reference number 20-APHM-01). The study was conducted according to the standards set out in the 1983 Declaration of Helsinki. Written informed consent to participate was obtained for all participants.

### Perioperative AF Monitoring

After cardiac surgery, patients were continuously monitored with telemetry in the intensive care unit for a minimum of 2 days. Subsequently, a daily electrocardiogram was systematically performed in conventional and recovery wards and at any time when patients experienced symptoms potentially related to a cardiac arrhythmia.

According to the latest European Society of Cardiology guidelines (19), paroxysmal AF was considered when episodes of AF terminated spontaneously or with intervention within 7 days of onset, persistent AF was considered when episodes of AF were continuously sustained beyond 7 days, and long-standing AF was considered when episodes of AF lasted longer than 1 year. Permanent AF was considered when AF was accepted by the patient and the physician and no further attempts to restore sinus rhythm were undertaken. Permanent AF was not included in this study. All patients not in sinus rhythm at the end of cardiac surgery had electrical cardioversion to restore sinus rhythm. PeOpAF was defined as an episode of AF exceeding 30 s, with or without related symptoms, regardless of whether they justified medical or electrical cardioversion, and which occurred during the postoperative hospitalization period.

### Measurement of BAC

Blood samples amounting to 3 ml for adenosine measurement were collected from patients during cardiac surgery, before starting extracorporeal circulation, as previously described (16). Blood samples were collected for patients and controls using a stop solution as previously described (17). BAC was then evaluated by liquid chromatography/mass spectrometry (LC/MS-MS, Shimatzu, Marne la vallée, France) as previously described (6). The detection threshold was 1 nM and the intra assay coefficient of variation was < 10%.

### Preparation of PBMC

Peripheral blood mononuclear cells were isolated from the blood using the Vacutainer CPT™ system (Becton-Dickinson^®^, Rungis, France) following venipuncture from the brachial vein according to the instructions of the manufacturer. Briefly, blood samples were centrifuged within 2 h of collection. After centrifugation, mononuclear cells were collected from the plasma/Ficoll interface and washed twice with a phosphate buffer solution (PBS). The cell pellet was stored at −80°C until used for western blot analysis.

### Cardiac Tissue Collection

A small fragment of atrial tissue was removed at the site of the extracorporeal circulation intake puncture and the site of the left atriotomy when surgically necessary. Samples (~1 mm^3^; median weight 1.1 mg, range 0.8–1.4 mg) were taken using a cold knife blade to preserve tissue integrity. We considered that such low-volume samples would not affect the quality and size of the atrium scar at the end of the procedure, or the length of surgery, and would provide sufficient material to address adenosine receptor expression. Tissue samples were stored in liquid nitrogen. Fresh tissue samples from the atria were tested extemporaneously in cAMP assays.

### Western Blot

The procedure for western blotting has been described (16, 17, 20, 21). Briefly, A_1_R, A_2A_R, and A_2B_R expression in PBMCs and atrium tissues were determined by western blot using Adonis, an agonist-like monoclonal antibody to human A_2A_R (22), a rabbit polyclonal antibody to human A_1_R (ab82477, Abcam^®^, Paris, France), and a goat polyclonal antibody to human A_2B_R (ab40002, Abcam^®^, Paris, France). PBMC pellets (0.25 × 10^6^ cells) and tissue samples (0.5 mg) were solubilized using lysis buffer and sonication. Samples were then submitted to standard 12% polyacrylamide gel electrophoresis under reducing conditions before transfer to a polyvinylidene difluoride membrane. The filter was then incubated with Adonis (1 μg/ml) or A_1_R or A_2B_R antibodies (0.25 μg/ml). Blots were revealed using phosphatase alkaline-labeled anti-species second antibodies and *ad hoc* colorimetric substrate. Each band corresponding to the adenosine receptors (36 kDa for A_1_R, 45 kDa for A_2A_R, and 37 kDa for A_2B_R) was submitted to densitometry analysis using the Image J 1.42q software (National Institutes of Health). Results are expressed in arbitrary units (AU), defined as the ratio of pixels generated by the adenosine receptor band to pixels generated by the background signal, as previously described (20, 21). In these conditions, the intra- or the inter-assay coefficient of variability was < 10%.

### Statistical Analysis

Patient data are expressed as *M* ± *SD* or median with interquartile range. Since different groups of patients can be considered independent, comparisons of quantitative variables were performed using the Mann-Whitney U tests. Correlations between adenosine receptor production in PBMCs and the atria were quantified and tested using Pearson's correlation coefficient. All statistical tests were two-sided and *P-*values <0.05 were considered statistically significant. The analysis was performed with the GraphPad Prism program version 8.4.3.

## Results

The study group consisted of 39 patients (30 men and 9 women; mean age 65 years, range 40–82 years) undergoing cardiac surgery, among which 24 patients undergoing mitral valve surgery (14 mitral valve repairs, 8 mitral valve replacements, and 2 LA cardiac myxoma resections) and 15 undergoing coronary artery bypass surgery (who were included in a previous study 16). Among the patients who underwent mitral valve surgery, 19 had a severe mitral valve regurgitation (mean regurgitation orifice area = 52.1 ± 8.1 mm^2^ and mean regurgitation volume = 77.2 ± 11.3), and 3 others had severe mitral valve stenosis (mean mitral valve gradient 15.7 ± 4.2 mmHg). Furthermore, 15 (38%) patients experienced PeOpAF (6 of whom had AF before surgery, in which 3 had paroxysmal and 3 had persistent AF), while 9 experienced only *post-op* AF (3 paroxystic and 6 persistent). The clinical characteristics of the patients, according to the presence or absence of PeOpAF, are detailed in Table 1. With the exception of age and sex, patients with and without PeOpAF were similar in terms of the usual predictors of AF. None of the patients had a cardiovascular event during surgery or hospitalization. The control population comprised 20 healthy subjects (8 women and 12 men; mean age 60 years, range 39–72).

**Table 1:** Demographic characteristics of patients undergoing cardiac surgery.

**Variable**	**PeOpAF (*n* = 15)**	**No PeOpAF (*n* = 24)**	***P* value**
Female sex n (%)	6 (40)	3 (12)	0.05
Mean age (years)	70.9 ± 9.1	60.5 ± 9.7	0.02
**Cardiovascular risk factor**			
Hypertension	6 (40)	10 (42)	0.92
Dyslipidemia	6 (40)	12 (50)	0.54
Diabetes mellitus	1 (7)	2 (8.3)	0.85
Peripheral arteriopathy	2 (13)	0 (0)	
**Underlying cardiac surgery**			
Mitral valve surgery	11 (73)	11 (46)	0.09
Coronary bypass	4 (27)	12 (50)	0.15
Mixome	2 (13)	0	
**Echocardiography**			
LVEF (%)	59.5 ± 7.9	59.5 ± 12.3	0.98
Significant LVEF impairment	1 (7)	4 (17)	0.36
Left atrium size(mL/m^2^)	55.2 ± 23.1	44.3 ± 23.9	0.17
Creatinine concentration (μM)	94.3 ± 28.0	87.6 ± 17.9	0.36
**Cardiac medication**			
Aspirin	4 (27)	10 (42)	0.34
Clopidogrel	3 (20)	4 (17)	0.79
Beta-blocker	5 (33)	12 (50)	0.31
Statin	6 (40)	12 (50)	0.15
Fibrate	1 (7)	0 (0)	
Angiotensin converting enzyme inhibitor	7 (47)	11 (45.846)	0.96
Calcium inhibitor	4 (27)	2 (8.3)	0.12
Amiodarone	3 (20)	0 (0)	
Vitamin K inhibitor	2 (13)	0 (0)	
Direct oral anticoagulant	3 (20)	0 (0)	

*LVEF, left ventricular ejection fraction; PeOpAF, perioperative atrial fibrillation; SD, standard deviation*.

### Blood Adenosine Concentration (BAC)

**Figure 1:**
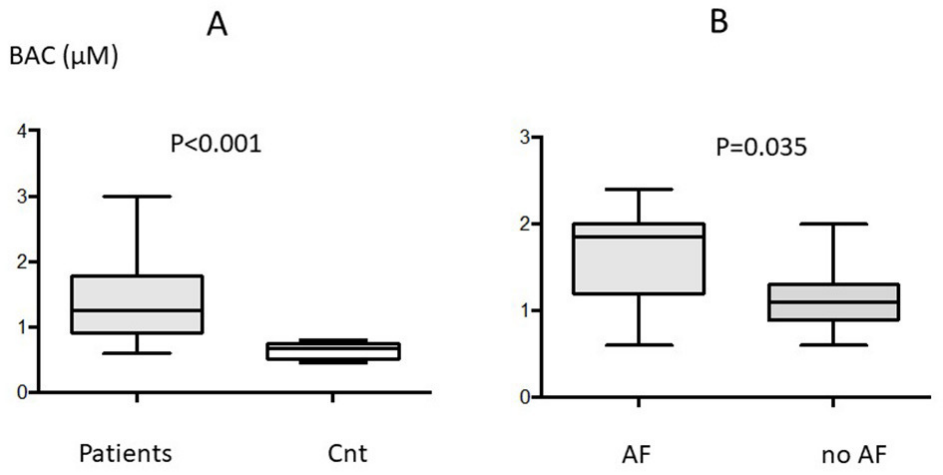
Blood adenosine concentration (BAC) in the whole population (*n* = 39) of patients **(A)** and in controls; and **(B)** in patients with (*n* = 15) or without (*n* = 24) perioperative atrial fibrillation (AF). Box indicates mean ± *SD* and vertical lines indicate the range.

Mean peripheral BAC was higher in patients vs. controls (1.33 ± 0.56 vs. 0.63 ± 0.11 μM; *p* < *0.0*01; Figure 1A). Among patients, mean peripheral BAC was higher in those with vs. without PeOpAF (1.72 ± 0.62 vs. 1.09 ± 0.34; *P* = 0.035 Figure 1B).

No significant differences were found between patients with pre- and post-operative AF vs. those with only post-operative AF.

Finally, no significant differences appeared between patients with mitral pathology vs. others (1.34 ± 0.5 μM vs. 1.23 ± 0.5 μM; *p* = 0.37).

### Adenosine Receptor Production

The production of A_1_R in the LA and right atrium (RA) could be analyzed in all 39 cases. A_2A_R production could be analyzed in 28 cases for the RA and in 39 cases for the LA, and A_2B_R production in 26 and 33 cases, respectively.

#### PBMCs

**Figure 2:**
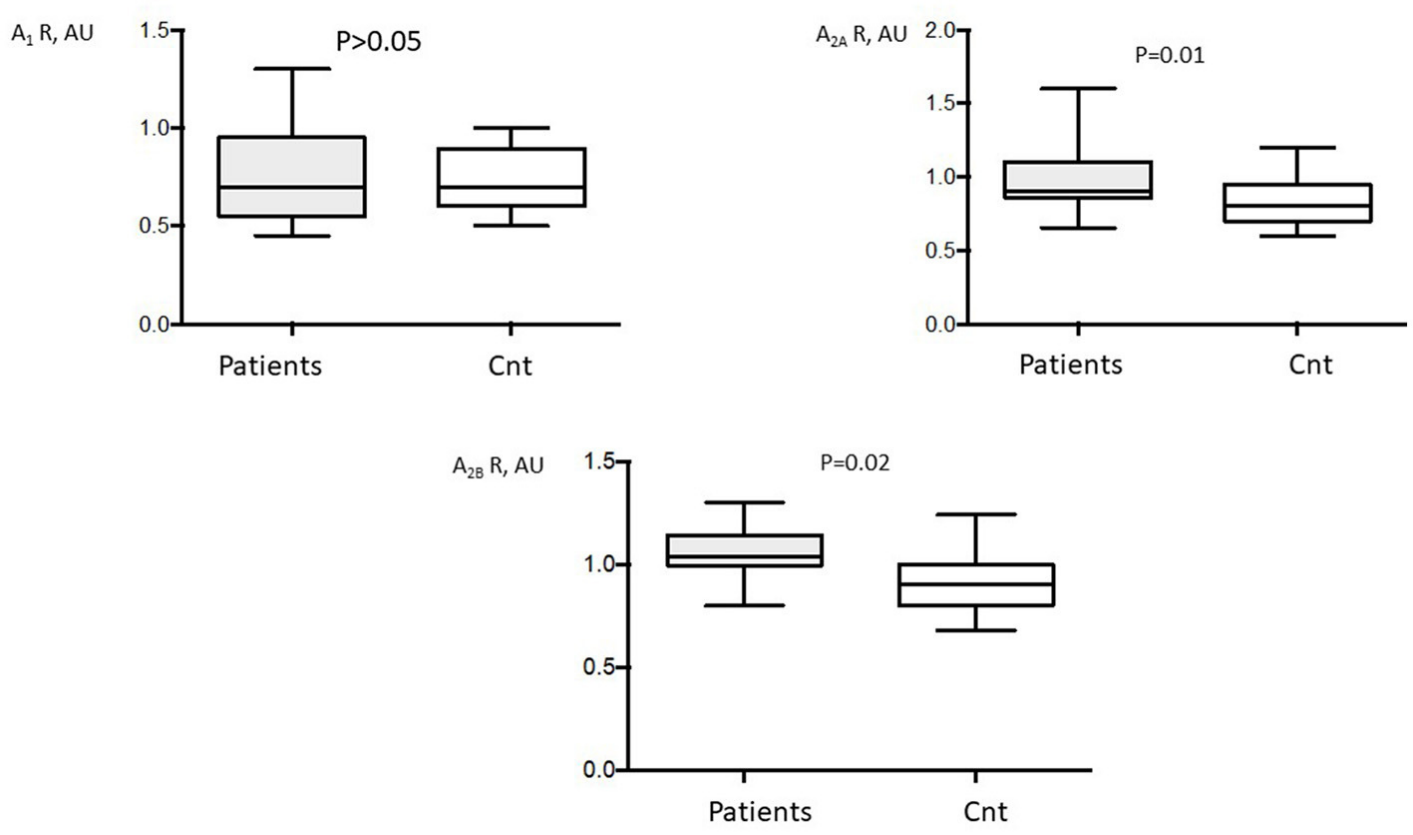
Adenosine receptor (AR) production in A_1_ (A_1_R), A_2A_, (A_2A_R), and A_2B_ (A_2B_R) adenosine receptors of patients undergoing cardiac surgery (*n* = 39). Production of adenosine receptors was performed by western blot in peripheral blood mononuclear cells (PBMCs). Control western blotting was performed on PBMC from 20 control subjects (Cnt). AU, arbitrary units. Box indicates mean ± *SD* and vertical lines indicate the range.

**Figure 3:**
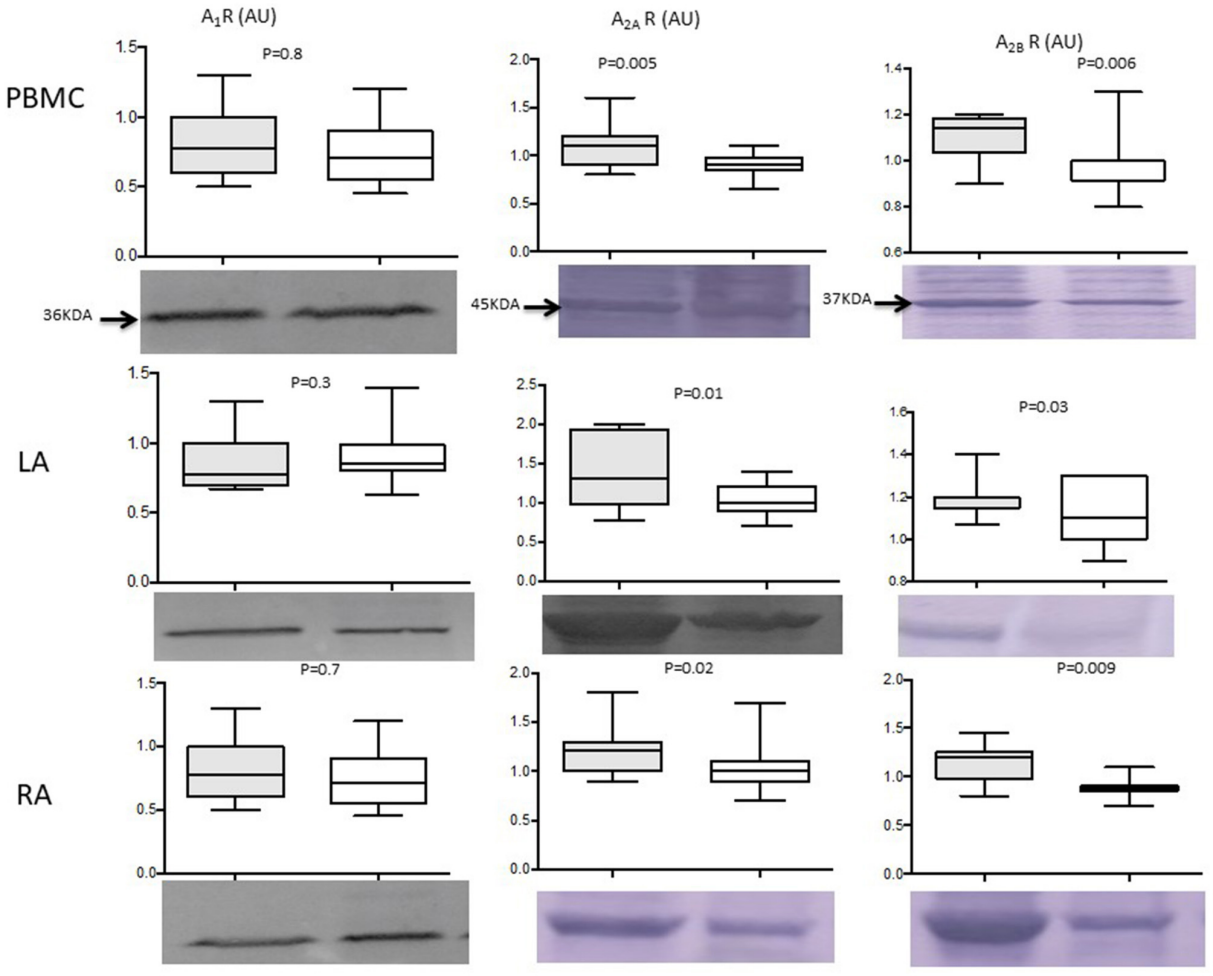
Central picture: Adenosine receptor production evaluated by western blotting, in 39 patients undergoing cardiac surgery [15 with perioperative atrial fibrillation (PeOpAF) in the gray box]. White box represents patients without perioperative atrial fibrillation. PBMCs, peripheral blood mononuclear cells; LA, left atria; RA, right atria; KDA, molecular weight in kilodaltons. A_1_ R, A_2A_R, and A_2B_ R are A_1_, A_2A_, and A_2B_ adenosine receptors respectively. Box indicates mean ± *SD* and vertical lines indicate the range. Adenosine receptor production in PeOpAF patients (gray box) vs. no PeOpAF (white box).

No significant difference was observed in A_1_R production in patients vs. controls (0.76 ± 0.22 vs. 0.73 ± 0.15; *P* >0.05, Figure 2) or between patients with and without PeOpAF (0.77 ± 0.22 vs. 0.76 ± 0.23, respectively; *P* >0.05; Figure 3). In contrast, patients showed significant overproduction of A_2A_R (47%) (0.97 ± 0.17 vs. 0.66 ± 0.15; *P* = 0.01 and A_2B_R (13.6%) (1 ± 0.13 vs. 0.88 ± 0.12; *P* = 0.02; Figure 2) compared with controls.

Patients with PeOpAF vs. those without PeOpAF showed a higher production of A_2A_R (25%; 1.12 ± 0.21 vs. 0.89 ± 0.1; *P* = 0.005) and A_2B_R (14.5%) (1.1 ± 0.1 vs. 0.96 ± 0.12; *P* = 0.006) (Figure 3). No significant differences were found between patients with pre- and post-operative AF vs those with only post-operative AF.

Finally, no significant difference appeared between patients with mitral pathology vs. others (A_1_R:0.74 ± 0.2 AU vs. 0.8 ± 0.3 AU; *p* = 0.27. A_2A_R:0.98 ± 0.23 AU vs. 0.96 ± 0.11 AU; *p* = 0.72. A_2B_ R: 1.05 ± 0.09 AU vs. 1.05 ± 0.2 AU, *p* = 0.8).

#### Atria

The production of A_2A_R and A_2B_R was higher in patients with PeOpAF (Figure 3), whereas no significant difference was found in A_1_R production. A_2A_R: LA: 1.37 ± 0.44 vs. 1.03 ± 0.19 (+33%), *p* = 0.01. RA: 1.21 ± 0.25 vs. 1.03 ± 0.24 (+17%, *p* = 0.02). A_2B_R: LA: 1.2 ± 0.09 vs. 1.09 ± 0.13, (+10% *p* = 0.03). RA: 1.12 ± 0.18 vs. 0.91 ± 0.11 (+23%, *p* = 0.009). Finally, no significant difference was found in A_1_R production between the LA and RA, whereas there was a gradient in A_2_R production between the RA and LA, with A_2A_R and A_2B_R production higher, at 12% and 15%, respectively, in the LA (1.11 ± 0.25 vs. 0.99 ± 0.23 and *P* = 0.02; 1.13 ± 0.1 vs. 0.98 ± 0.18; *P* = 0.009, respectively). No significant differences were found between patients with pre- and post-operative AF vs. those with only post-operative AF, concerning adenosine receptors production. In addition, no significant difference appeared between patients with mitral pathology vs. others. A_1_R, LA: 1.25 ± 0.4 AU vs. 0.82 ± 0.3 AU; *p* = 0.9. RA:0.87 ± 0.3 vs. 0.95 ± 0.28; *p* = 0.5.

A_2A_R, LA: 1.13 ± 0.19 AU vs. 1.05 ± 0.09 ± 0.11 AU; *p* = 0.8. RA: 1.12 ± 0.3 vs. 1.09 ± 0.18, *p* = 0.7. A_2B_R, LA: 1.05 ± 0.09 AU vs. 1.05 ± 0.2 AU, *p* = 0.8. RA: 1.16 ± 0.09 vs. 1.25 ± 0.12, *p* = 0.14.

#### Correlation Between PBMC, LA, and RA Adenosine Receptor Expression

**Figure 4:**
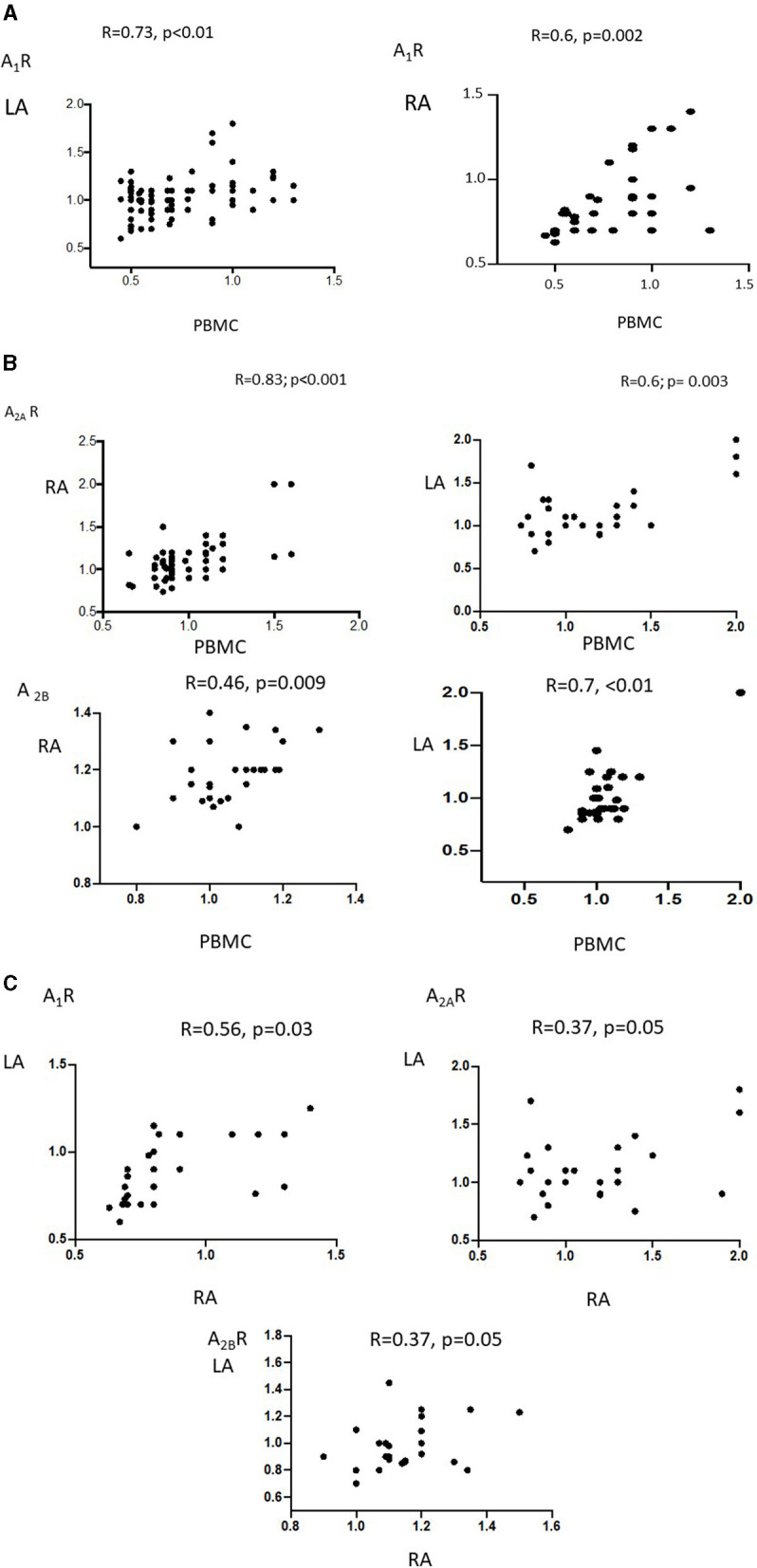
Pearson's R correlation between adenosine receptor expression by PBMCs in the LA (left panel) and RA (right panel) in patients undergoing cardiac surgery: **(A)** A_1_R, **(B)** A_2A_R and A_2B_R. In **(C)** the correlation between adenosine receptors subtypes in RA vs. LA.

A_1_R, A_2A_R, and A_2B_R production by PBMCs correlated with the expression of these receptors in the RA and LA (Figures 4A,B). A_1_R, A_2A_R, and A_2B_R production in the LA and RA were correlated (R = 0.56, *P* = 0.03 for A_1_R; and R = 0.37, *P* = 0.05 for A_2A_R and A_2B_R; Figure 4C). Finally, no correlation was observed between BAC and adenosine receptors production.

## Discussion

This study evaluates adenosine receptor expression in both PBMC, RA, and LA. It showed that patients with structural heart disease, who underwent cardiac surgery had higher BAC and higher adenosine A_2A_R and A_2B_R expression in the atria and PBMCs when they experienced PeOpAF rather than continuous sinus rhythm. Furthermore, in the patient population as a whole, we observed a gradient of A_2_R levels, with higher A_2A_R and A_2B_R expression in the LA than in the RA. No statistically significant difference in A_1_R expression in the PMBCs, LA, or RA was found between patients and controls or between patients with and without PeOpAF. We observed a correlation between adenosine receptor expression in PBMCs and atria regardless of the subtype considered. These data also confirmed that PBMCs are a useful model for studying the adenosinergic system of AF patients (23). Finally, we do not observe a significant correlation between BAC and LA receptor production. Nonetheless, because receptors expression was high we could not exclude that receptors expression reached a plateau.

Previous studies have suggested that adenosine release is involved in PeOpAF (7, 24). In AF patients without structural heart disease, exogenous adenosine injection may frequently induce episodes of AF and pulmonary vein ectopy (5, 25) by shortening the duration and refractoriness of the action potential (22, 26). Both A_1_R and A_2A_R have been suspected to be involved in AF. Inhibition of the A_1_R pathway prevents the occurrence of AF in animals (27). A study involving optical mapping and immunoblots showed a concentration gradient (RA-to-LA) of G-protein, coupled inwardly rectifying potassium channels linked to adenosine A_1_R in the atria of explanted human hearts, leading to a greater RA vs. LA repolarization sensitivity in response to adenosine perfusion (28). In the present study, no significant overproduction of A_1_R was observed in our population. However, one should bear in mind that the transplanted heart, which may reflect an advanced heart failure mechanism of AF, is not representative of our population, as most of our patients exhibited a normal LVEF.

Furthermore, A_2A_R has also been suspected to be involved in AF (29). Thus, an increase in A_2A_R expression in the RA of patients with AF was associated with a high baseline level of spontaneous sarcoplasmic reticulum calcium release in myocytes. Moreover, activation of these receptors leads to the activation of ryanodine receptors, which control part of the intracellular calcium flux from the sarcoplasmic storage site (29). Together, these data strongly suggested the involvement of A_2A_R in the occurrence of AF in patients with underlying heart disease undergoing cardiac surgery. According to these results, our study is the first to show A_2A_R overproduction in LA.

It is well established that cardiac fibrosis participates in the progression of AF (30). All adenosine receptor subtypes have been suspected to be implicated in cardiac fibrosis modulation [for review see (31)]. A_1_R activation by agonists led to a decrease in interstitial fibrosis, and overexpression of A_1_R protected against the remodeling process after an experimental myocardial infarct. However, here we did not observe a significant change in A_1_ R production in AF patients.

While A_2A_R has likely anti-fibrosis properties in the heart due to its anti-inflammatory properties (4, 31), the implications of A_2B_R in fibrosis remain controversial (31). A_2B_R overproduction has been associated with a decrease in cardiac collagen and protein synthesis (32). These effects were secondary to cAMP production as a transduction signal pathway (33). However, whereas most of the *in vitro* studies seemed to indicate an antifibrotic action of A_2B_R on cardiac tissues, more recent *in vivo* studies seemed to indicate that the blockage of A_2B_R may be beneficial. Thus, it was shown that the use of a specific A_2B_R antagonist inhibits fibrosis in experimental myocardial infarction (34). This result was confirmed using knockout mice for A_2B_R, demonstrating that A_2B_R contributed to post-infarctus heart failure (35). Finally, selective blockade of A_2B_R inhibited caspase 1 activity and leucocyte infiltrate (36), and attenuated secretion of profibrotic and proinflammatory mediators such as transforming growth factor-beta, tumor necrosis factor-alpha, and interleukin-6 (36, 37). Here, we found overexpression of both A_2A_ and A_2B_ receptors in the atria. To the best of our knowledge, there was no data on the A_2B_ production level in perioperative AF. While the increase in A_2B_ production remains weak, this A_2B_ overproduction over a long period may promote atrial fibrosis (30) which is well known to participate in the pathophysiology of AF (38). However, the validation of this hypothesis requires further investigations.

## Limitations

We enrolled a population-based group of patients, but they showed dissimilarities in terms of mean age and sex, which may have favored PeOpAF. However, BAC and adenosine receptor expression is not known to be related to either, so these differences are unlikely to have influenced the main results. The population also showed different underlying cardiopathy. However, all included patients presented underlying structural heart disease which was well known to be associated with AF by favoring atrial cardiomyopathy (13). A different, continuous, and more prolonged AF screening protocol may have also been designed in our study. Therefore, short episodes of asymptomatic PeOpAF, occurring after the intensive care unit discharge, may have remained unnoticed, and thus this may have been responsible for information biases. However, this limitation is common with all studies looking for atrial fibrillation screening (39).

One could also emphasize that we enrolled patients with AF before surgery. Once again, increased risk of long-term stroke and the four to five-fold increased risk of recurrent AF in the next 5 years in the case of PeOpAF (12) suggests a close mechanism with non-PeOpAF (14) and underlying atrial cardiomyopathy (13). In that way, no statistical difference was found in any adenosine receptor production between patients with pre- and post-operative AF and patients with AF only after surgery.

Our study involved a relatively small sample of patients but is comparable with other surgical studies on adenosine receptor expression (28, 29). As the amount of cardiac tissue resected was small, we were unable to measure receptor activity, and can only suggest an association with RA and LA hyperactivity.

## Conclusion

Perioperative AF was associated with high BAC and high A_2A_R and A_2B_R expression, especially in the LA, after cardiac surgery in patients with underlying structural heart disease. Whether these increases favor the triggering of AF in this patient population needs further investigation.

## Data Availability Statement

The raw data supporting the conclusions of this article will be made available by the authors, without undue reservation.

## Ethics Statement

The studies involving human participants were reviewed and approved by CPP Sud Méditerranée, Marseille, France, Reference Number 20-APHM-01. The patients/participants provided their written informed consent to participate in this study.

## Author Contributions

BM: conceptualization, investigation, data curation, methodology, writing—original draft, and writing—review and editing. JF: conceptualization, formal analysis, methodology, writing—original draft, and writing—review and editing. CG, SL, CC, MCC, MC, MG, and JR: investigation and writing—review and editing. MM and FB: investigation, data curation, and writing—review and editing. FF and J-CD: conceptualization and writing—review and editing. VG: data curation, methodology, and writing—review and editing. GM: conceptualization, investigation, and writing—review and editing. RG: conceptualization, data curation, methodology, formal analysis, and writing—review and editing. All authors contributed to the article and approved the submitted version.

## Conflict of Interest

The authors declare that the research was conducted in the absence of any commercial or financial relationships that could be construed as a potential conflict of interest.

## Publisher's Note

All claims expressed in this article are solely those of the authors and do not necessarily represent those of their affiliated organizations, or those of the publisher, the editors and the reviewers. Any product that may be evaluated in this article, or claim that may be made by its manufacturer, is not guaranteed or endorsed by the publisher.
